# Two case reports of rare cocaine-associated dermatoses: Pyoderma gangrenosum and linear IgA bullous dermatosis

**DOI:** 10.1177/2050313X251350333

**Published:** 2025-06-20

**Authors:** Daniela Cino, Barbara Marzario, Sophia Colantonio, Mark G. Kirchhof

**Affiliations:** 1 Division of Dermatology, Department of Medicine, University of Ottawa, ON, Canada; 2 The Ottawa Hospital, ON, Canada

**Keywords:** cocaine, dermatoses, pyoderma gangrenosum, linear IgA bullous dermatosis

## Abstract

We report two cases of cocaine-associated dermatoses: pyoderma gangrenosum and linear IgA bullous dermatosis. These cases highlight the importance of considering less common associations with drug use in the development of certain dermatoses. Eliciting a good history around the onset of the skin condition and potential triggers is vital in establishing a causal link. In both of our cases, the lesions improved with cocaine avoidance and disease-specific treatment. Use of cocaine is most commonly associated with vasculitis, but has been reported to trigger neutrophilic dermatoses and vesiculobullous diseases. Dermatologists need to be aware of these less common cocaine-associated dermatoses, particularly when patients are not responding to standard therapy alone.

## Introduction

Cocaine use in Canada has increased in prevalence.^1^ Furthermore, based on reports, up to 80% of cocaine is adulterated with levamisole, an anthelminthic agent.^2,3^ Cocaine use is associated with cutaneous manifestations including vasculitis, and less frequently with neutrophilic dermatoses and vesiculobullous diseases.^4^ As such, dermatologists need to consider cocaine in the development of certain skin diseases, particularly if there is noted treatment resistance to standard therapy. We report two cases of cocaine-associated dermatoses, one patient with pyoderma gangrenosum (PG) and the other with linear IgA bullous dermatosis (LABD), that improved with cocaine avoidance and standard therapy treatment.

PG is a neutrophilic dermatosis characterized by a painful nodule or pustule evolving into a deep ulcer, often with violaceous undermined edges. Approximately 50% of cases have an underlying etiology such as inflammatory bowel disease, rheumatoid arthritis, and hematologic malignancy.^5,6^ Another well-known trigger for PG is cocaine contaminated with levamisole.^3,7^ Levamisole-contamina-ted cocaine has also been implicated in leukocytoclastic vasculitis, thrombotic vasculopathy, and cutaneous necro-tizing vasculitis.^8^ We present a case of treatment-resistant cocaine-induced PG successfully treated with ustekinumab and cocaine avoidance.

LABD is a rare immune-mediated disease with variable clinical morphology. Drug-induced LABD presents with widespread vesiculobullous lesions with macular erythema. Less commonly, it can present as a morbilliform, localized, erythema multiforme, or Stevens–Johnson-like eruption. The most common medication culprit is vancomycin, but others have been reported.^9^ We present the second case of cocaine-induced LABD successfully treated with dapsone and cocaine avoidance.

## Case report

### Case 1

**Figure 1.**
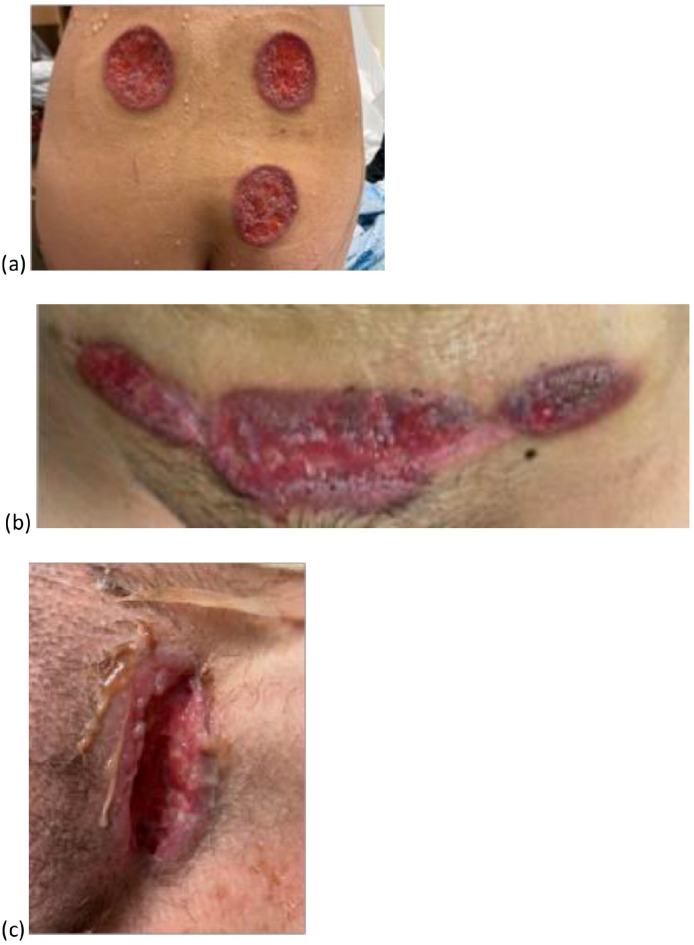
On the lower back, buttock (a), and cesarian section scar (b), there are annular ulcers with a purulent base with an undermined and overhanging, gunmetal-colored border. (c) In the left genitocrural fold, there is a knife-like ulceration with a purulent base, however, no inflammatory border.

A 36-year-old female presented with a 3-year history of recalcitrant cutaneous ulcers. She had multiple annular cribriform ulcers on the back, buttocks, breasts, and C-section scar that were clinically in keeping with PG (Figure 1(a) and (b)). She also had deep, linear, knife-like ulcers without undermined edges or inflammatory borders in the left genitocrural fold. This lesion was morphologically dissimilar to her other lesions and clinically in keeping with cutaneous Crohn’s disease (Figure 1(c)). She had a saddle nose deformity and reported a remote history of cocaine use; however, she denied current use initially.

An extensive workup was performed. Infectious serologies were negative for HIV, hepatitis B and C, and syphilis. A thorough autoimmune workup revealed negative antinuclear antibodies, negative double-stranded deoxyribonucleic acid, negative rheumatoid factor, and inconclusive antineutrophil cytoplasmic antibody (ANCAs). Of note, previous workup had revealed a positive cytoplasmic ANCA (c-ANCA) and proteinase 3 (PR3) of 21. Tissue culture was also negative. She had a normal colonoscopy. Skin biopsy of the right shoulder showed mixed inflammation with prominent plasma cells, lymphocytes, and aggregates of neutrophils, which was compatible with PG. Skin biopsy of the left genitocrural fold ulcer was also consistent with PG. Urine toxicology was positive for cocaine. Overall, her clinical presentation correlated well with other cocaine-induced PG cases in the literature.

Her PG was refractory to numerous topical and systemic treatments. She received multiple courses of antibiotics, including doxycycline, clindamycin, cephalexin, and metronidazole. Topicals that were trialed without success included tacrolimus, crisaborole, and clobetasol. She did not respond to colchicine, dapsone, and cyclosporine. She responded to repeated tapering courses of prednisone 40–50 mg and was transitioned to intravenous immunoglobulin, at which time she achieved complete remission of her PG. For maintenance, she was started on ustekinumab 45 mg every 8 weeks. With cocaine avoidance and ustekinumab, she has remained in remission for over 2 years.

### Case 2

**Figure 2.**
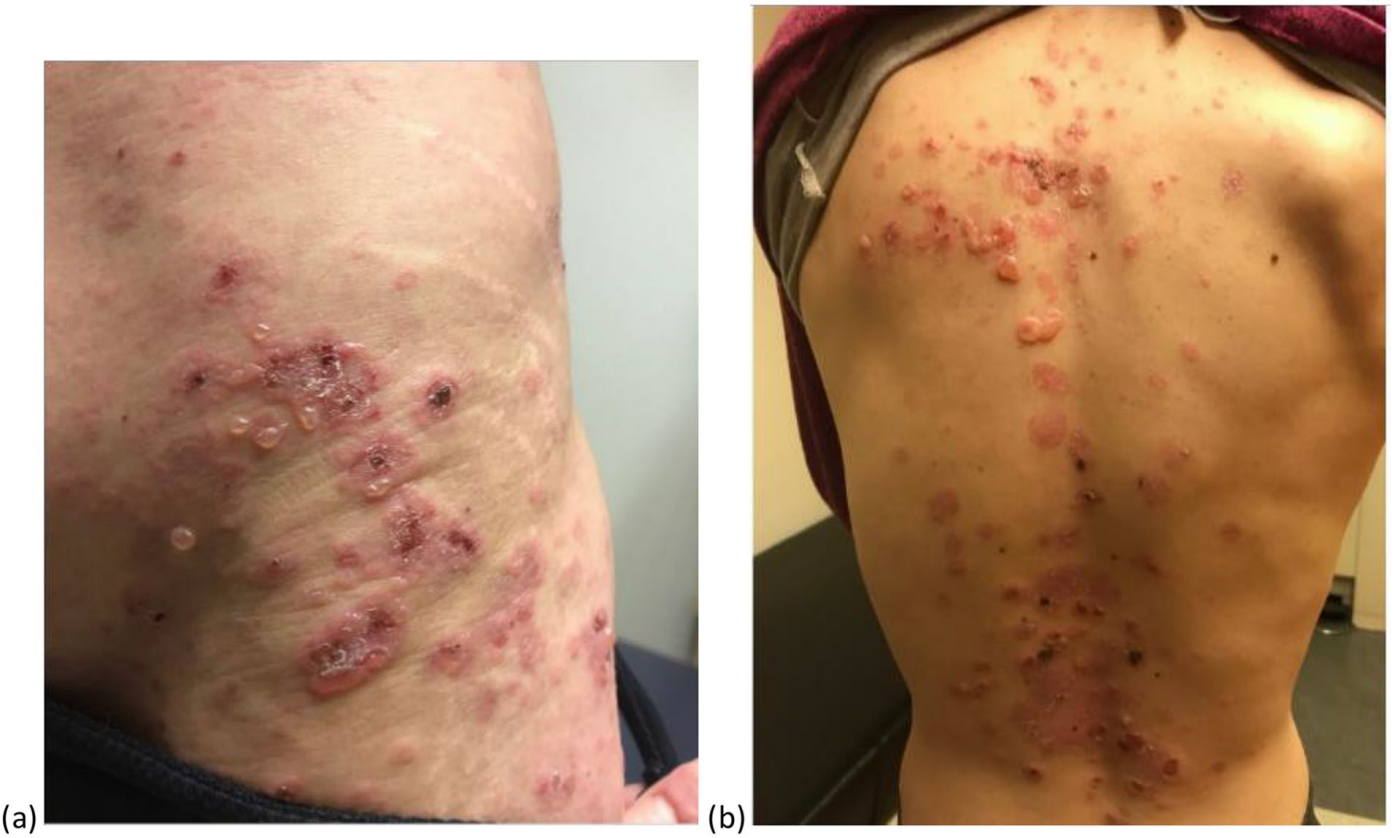
On the flank and hip (a) and on the back (b), there are several groups of vesicles and bullae in an annular arrangement with central clearing and superficial erosions with hemorrhagic erosions.

A 36-year-old female with a history of juvenile rheumatoid arthritis presented with episodes of recurrent vesicles and bullae on the face, scalp, trunk, and extremities that partially responded to antibiotics. The vesiculobullous lesions were in an annular arrangement with central clearing (Figure 2(a) and (b)). The patient was diagnosed clinically with LABD, confirmed by skin biopsy, which demonstrated subepidermal blisters with neutrophils, and direct immunofluorescence, which was positive for IgA linearly along the dermal–epidermal junction. She was treated with prednisone, dapsone, and mycophenolate mofetil; however, there were challenges with treatment adherence. Review of previous lab results revealed positive cocaine metabolites coinciding with the initial presentation. She was counseled on cocaine avoidance to improve treatment results. Subsequently, she had improvement with cocaine avoidance and adherence to therapy of dapsone 50 mg daily. To our knowledge, there is only one other reported case of cocaine-induced LABD.^10^

## Discussion

Patients with cocaine-induced PG typically have a temporal relation between starting cocaine and worsening or relapse of ulcers.^8^ Lesions typically arise 1–4 weeks after initiation of cocaine use.^3^ PG is a unique manifestation of levamisole-contaminated cocaine, which is different from the typical levamisole vasculopathic eruptions. Patients with levamisole-induced vasculitis can have unusual serology findings. Perinuclear ANCAs (p-ANCAs) are present in more than 80% of cases, and c-ANCAs in about 50%.^11^ Some cases of levamisole exposure have shown discordant ANCAs with p-ANCA-positive patients having positive anti-PR3 antibodies, which usually are c-ANCA associated.^12^ Other cases include positive p-ANCA without antibodies against the typical target myeloperoxidase.^12^

This case is unique because clinically and histologically most of her cutaneous manifestations were in keeping with classic PG; however, her previous serologic findings (positive c-ANCA and PR3) that coincided with cocaine use reflect those seen in vasculitis. She had a recalcitrant course requiring multiple immunosuppressants, achieving remission with cocaine avoidance and ustekinumab, a monoclonal antibody targeting p40 subunit of interleukin-12 (IL-12) and IL-23. Patients with recalcitrant PG have increased expression of IL-23, and there have been case reports of successful use of ustekinumab in treatment-resistant PG.^13–18^ However, to our knowledge, this is the first cocaine-induced PG that has been treated successfully with ustekinumab.

Our second case is that of cocaine-induced LABD and is only the second report in the literature of this association.^10^ LABD is an autoimmune disease involving IgA deposition in the basement membrane, which leads to vesiculobullous lesions. Histologically, drug-induced LABD shows neutrophil-rich subepidermal blisters, which were seen in our patient. The lack of mucosal involvement and remission upon strict cocaine avoidance points toward drug-induced LABD. In both our case and the previously reported case, treatment with dapsone resulted in the resolution of the lesions.^10^

These two cases highlight the diversity of clinical presentations of cocaine-associated cutaneous disease. Dermatologists need to take a thorough history and consider screening for drug use, which is often required to confirm the diagnosis. Many patients will not feel comfortable disclosing their cocaine use due to fears of social and legal repercussions. In addition, we should consider a multidisciplinary approach to care and involve addiction services when warranted, as the mainstay of treatment is cocaine avoidance.
